# *Klebsiella pneumoniae* bioconjugate vaccine functional durability in mice

**DOI:** 10.1016/j.vaccine.2024.126536

**Published:** 2024-11-20

**Authors:** Paeton L. Wantuch, Cory J. Knoot, Emily C. Marino, Christian M. Harding, David A. Rosen

**Affiliations:** a Department of Pediatrics, Division of Infectious Diseases, Washington University School of Medicine, St. Louis, MO 63110, USA; b Omniose, St. Louis, MO 63110, USA; c Department of Molecular Microbiology, Washington University School of Medicine, St. Louis, MO 63110, USA

**Keywords:** *Klebsiella pneumoniae*, Vaccine, Bioconjugation, capsular polysaccharide, O-antigen polysaccharide, Serum bactericidal assay, Durability

## Abstract

*Klebsiella pneumoniae* is a leading cause of hospital-acquired infections as well as the leading cause of neonatal sepsis worldwide. Further, increasing antibiotic resistance in this pathogen makes *K. pneumoniae* troublesome to treat. Despite its clinical importance, there is not yet an approved *K. pneumoniae* vaccine available. Here we tested antibody durability and long-term functionality of two previously reported bioconjugate vaccines targeting the *K. pneumoniae* capsular type K2 and O-antigen type O1v1. We demonstrate that both antibodies are durable in mice for up to six months with significant IgG titers. However, only the K2 antibodies exhibit functionality out to six months as evidenced by serum bactericidal activity and survival in a murine bacteremia challenge model. These results are another promising step towards demonstrating the clinical capacity of bioconjugate vaccines and their induction of durable antibody responses.

## 1. Introduction

*Klebsiella pneumoniae* is a Gram-negative opportunistic pathogen responsible for causing human infections such as pneumonia, urinary tract infections, and sepsis [1]. Recently, *K. pneumoniae* was determined to be the most common etiologic pathogen contributing to deaths in children under the age of five in low- and middle-income countries [2,3]. Further, increasing antibiotic resistance mechanisms such as extended-spectrum beta-lactamases and carbapenemases in *K. pneumoniae* are resulting in infections that are extremely difficult to treat [4]. Therefore, alternative therapeutics and preventatives beyond antibiotics are desperately needed to combat this organism.

Immunization with polysaccharide-protein conjugate vaccines is an attractive preventative strategy that has been successfully implemented for other bacterial pathogens such as *Streptococcus pneumoniae*. More recently, a unique methodology, termed bioconjugation, has been implemented to synthesize conjugate vaccines using an engineered bacterial expression system. This allows for rapid production of diverse polysaccharide-protein conjugates. There are two major extracellular polysaccharides that serve as attractive candidates for a potential *K. pneumoniae* conjugate vaccine: the capsular polysaccharide (K-antigen) and the O-antigen polysaccharide of lipopolysaccharide (O-antigen). Our group and others have investigated both polysaccharides as components of conjugate vaccines demonstrating variable success in targeting *K. pneumoniae* [5,6]. However, studies have yet to be performed examining the longevity and durability of the immune response generated by these conjugate vaccines.

Herein we report on the longevity (antibody persistence over time) and durability (antibody functionality over time) of antibody responses from immunization with conjugate vaccines targeting the K2 K-antigen and O1v1 O-antigen. We previously reported on the production of these vaccines and the initial immunological response [5]. In that work we found both vaccines produce antibodies measured throughout the course of immunization up to two weeks post immunization. However, we found that despite the presence of both K2 and O1v1 antibodies, only the K2 antibodies were functional and able to protect mice [5]. In this current work we assess the antibody durability out to six-months post-vaccination measuring IgG titers and subtypes, antibody functionality, and survival in a murine bacteremia model. These findings corroborate the clinical importance of conjugate vaccines targeting *K. pneumoniae* and further establish their long-term immunologic response.

## 2. Methods

### 2.1. Bacterial strains and bioconjugate vaccine production

All bacterial strains used for this study were previously determined to be capsular type K2 and O-antigen type O1v1 [5,7]. Two of the strains used in this study are part of BEI Resourced Repository *Klebsiella pneumoniae* diversity panel (736214, 43816, and 511348) [7]. The *E. coli* strains used for bioconjugation production and subsequent assay coating were previously described [5]. K2-EPA and O1-EPA bioconjugates were produced and purified using methods previously described [5]. EPA is a genetically inactive mutant of Exotoxin A for *Pseudomonas aeruginosa* (EPA).

### 2.2. Murine vaccination

All murine immunizations complied with ethical regulations and standards for animal testing as set by the Washington University School of Medicine in St. Louis and the Institutional Animal Care and Use Committee at Washington University in St. Louis. Five-week-old male and female CD-1 mice (Charles River) were subcutaneously injected with 100 μL of either K2-EPA or O1-EPA adjuvanted with Alhydrogel 2 % aluminum hydroxide gel (InvivoGen) at a 1:9 ratio (50 μL vaccine to 5.5 μL adjuvant in 44.5 μL sterile PBS). All vaccination groups received 1 μg of polysaccharide based on total polysaccharide content as measured by anthrone-sulfuric acid assay. Mice were immunized on days 0, 14, and 28 and sera was collected on days 0, 42, 56, 70, 84, 98, 112, 126, 140, 154, 168, 182, and 196 following immunization.

### 2.3. Enzyme-linked immunosorbent assays

ELISA assays were completed as previously described [5]. Briefly, 96-well plates were coated overnight with ~10^6^ CFU/100 μL per well of the specified *K. pneumoniae* or *E. coli* isolate in sodium carbonate buffer. After coating, wells were blocked with 1 % BSA in sterile PBS followed by a wash with 0.05 % PBS-Tween-20. Mouse sera were diluted 1:100 and added to wells in triplicate for 1 h at room temperature. Anti-mouse-IgG-HRP (or specified IgG subtypes) was used as secondary at 1:5000 dilution. Plates were developed using 3,3^’^,5,5^’^-tetramethyl benzidine substrate and absorbance read at 450 using a microplate reader. Total IgG and IgG subtype concentrations were determined using a standard curve. All wells were normalized to blank wells treated the same as sample wells without receiving primary mouse sera. All graphs and statistics were generated using GraphPad prism 10.

### 2.4. Serum bactericidal assay

Serum bactericidal assays were carried out as previously described [5]. Briefly, *K. pneumoniae* isolates were diluted in sterile PBS at a 1:80,000 dilution. The assay mixture was prepared as 70 μL of bacteria, 20 μL heat-inactivated diluted mouse sera (1:20 final dilution), and 10 μL baby rabbit complement. Mouse sera was inactivated at 56 degrees for 30 min. Control wells were treated the same as samples except for receiving heat-inactivated diluted pre-immune serum. After the final incubation, samples were serially titrated and plated for colony enumeration. SBA titers were reported as percent survival compared to control wells. All graphs and statistics were generated using GraphPad Prism version 10.

### 2.5. Murine challenge experiments

Bacterial infections were administered via intraperitoneal injection. 43816 was grown statically in LB broth for 16 h at 37 degrees followed by centrifugation and resuspension in sterile PBS to OD_600_ ~ 1.0. Cultures were diluted further at 1:80,000 in sterile PBS with a respective challenge dose of 2500 CFU/ 50 μL. Mice were monitored for survival and weight loss daily for 7 days. Pairwise survival differences were determined by the Log-rank (Mantel-Cox) test. All graphs and statistics were generated using GraphPad Prism version 10.

## 3. Results

**Fig. 1.**
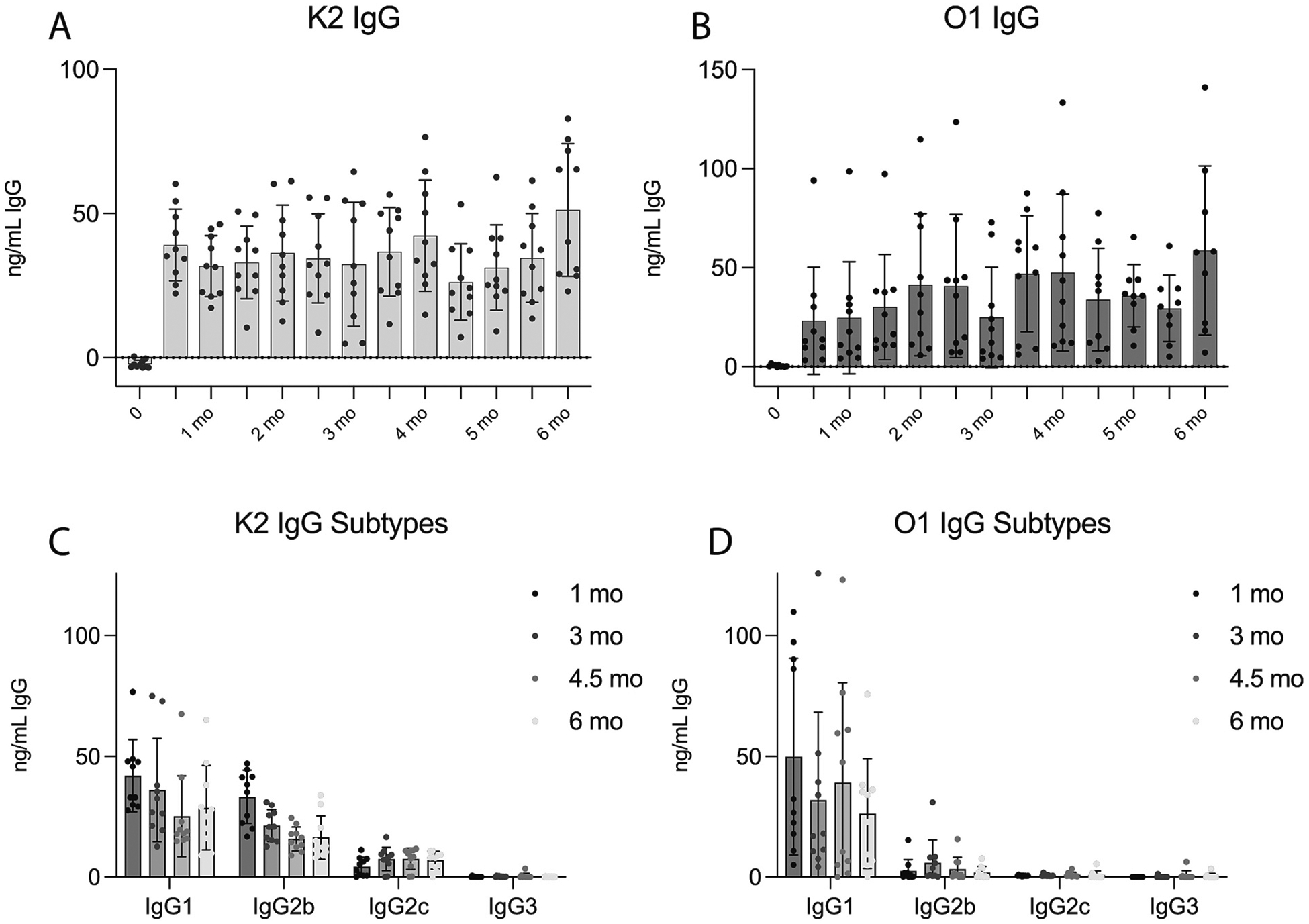
IgG concentrations generated from bioconjugate vaccines. Murine IgG kinetics after vaccination with either K2-EPA (A) or O1-EPA (B) over the course of six months as measured by ELISA against *E. coli* expressing the K2 *K. pneumoniae* capsular polysaccharide (A) or *E. coli* expressing the O1 O-antigen from *K. pneumoniae* (B). IgG subtypes were elicited after immunization with the K2-EPA bioconjugate (C) or O1-EPA bioconjugate (D) by ELISA using plates coated with the K2- or O1-expressing *E. coli*, respectively. Error bars represent standard deviation.

Male and female CD-1 outbred mice were immunized with bioconjugate vaccines targeting the K2 capsular polysaccharide or the O1 O-antigen polysaccharide. Mice were monitored and bled for serum every two weeks over a six-month period. Mouse sera was then analyzed using ELISA for K2- and O1-specific IgG titers. Plates were coated with *E. coli* that lacks its own capsule or LPS and was engineered to express either the K2 capsular polysaccharide or O1 O-antigen of *K. pneumoniae* as previously described [5]. All mice generated robust titers against both K2 and O1 that stayed consistent over the 6-month period with no statistically significant decrease in IgG concentration (Fig. 1A, B). Additionally, IgG subtypes were investigated. Mice immunized with K2-EPA generated significant IgG1 and IgG2b titers, small amount of IgG2c and no detectable IgG3 (Fig. 1C). Mice immunized with O1-EPA bioconjugates, however, only generated robust levels of IgG1, small levels of IgG2b, and no detectable IgG2c or IgG3 (Fig. 1D).

**Fig. 2.**
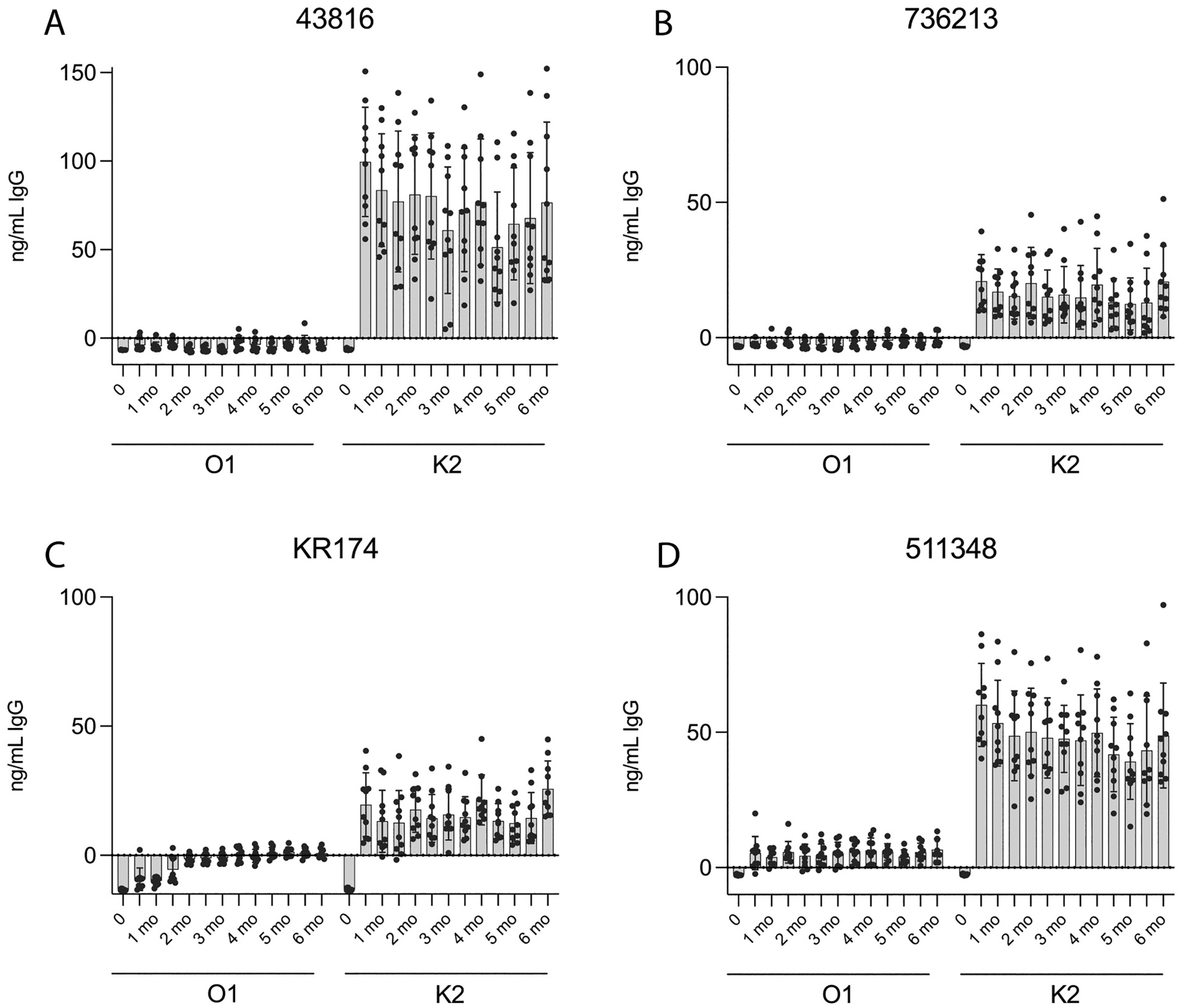
Detection of IgG binding to various K2:O1 strains of *K. pneumoniae*. K2- or O1-specific IgG titers were measured using plates coated with various *K. pneumoniae* strains that each express the K2 capsular polysaccharide and the O1 O-antigen: A) 43816, B) 736213, C) KR174, or D) 511348. Error bars represent standard deviation.

Next, we assessed the antibodies’ ability to bind various K2:O1-expressing isolates of *K. pneumoniae*. Plates were coated with either 43816, 736213, KR174, or 511348 isolates of *K. pneumoniae*. Similar to the *E. coli*-coated ELISAs, we observed robust IgG titers to the K2 capsule from K2-EPA-immunized mice against all four strains of *K. pneumoniae* (Fig. 2). There was not a statistical difference in IgG concentration against any of the four strains from 1 month to 6 months, demonstrating that the level of IgG remained consistent and persisted for 6 months. However, we observed little to no O1 titers in three of the four strains (Fig. 2A, B, C) with only very slight O1 titers observable against the 511348 strain (Fig. 2d). This is most likely due to the capsular polysaccharide shielding the O-antigen polysaccharide from antibody recognition, as has been previously demonstrated [5,6].

**Fig. 3.**
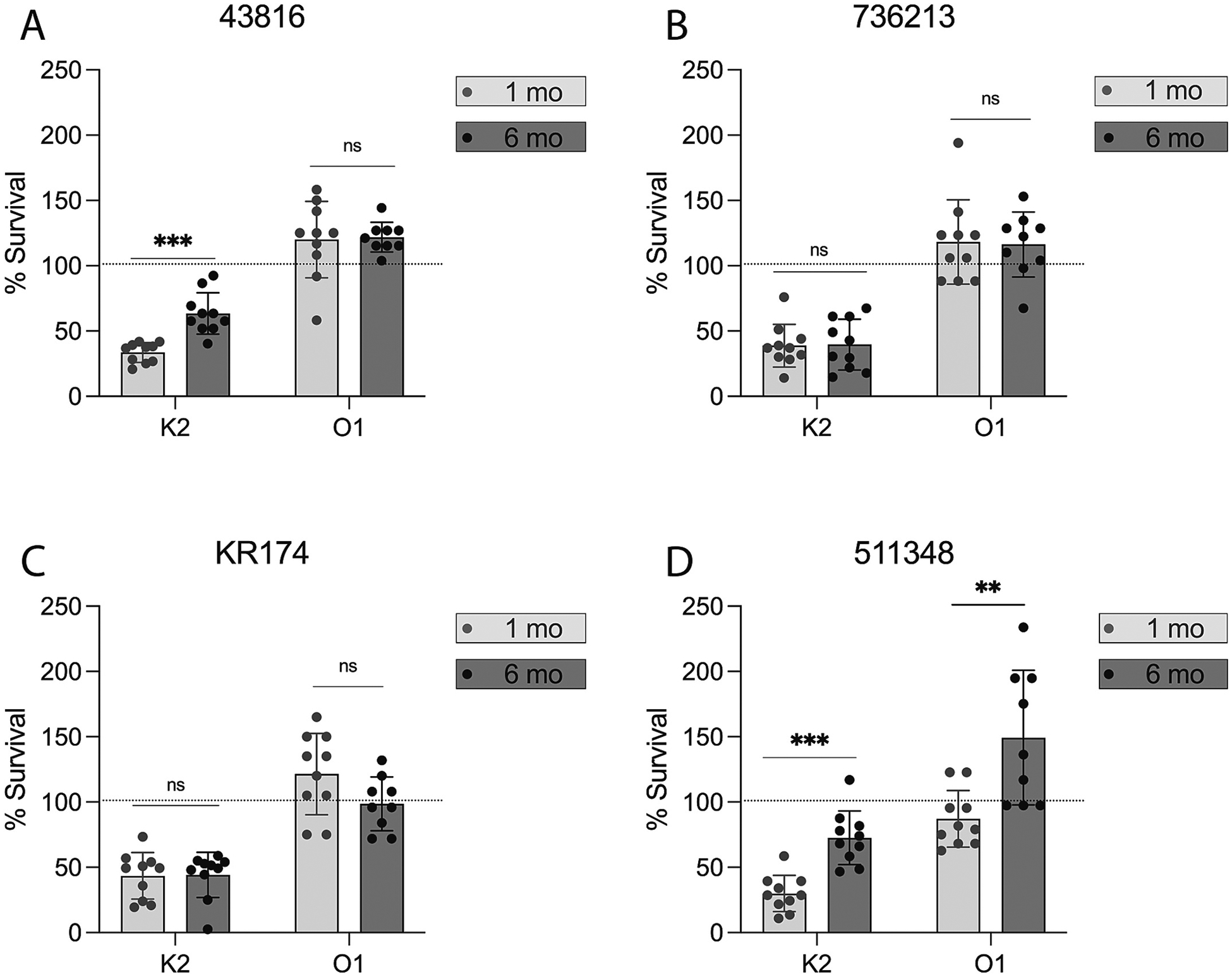
Serum bactericidal assays with vaccination mouse sera. Serum bactericidal activity of 1-month and 6-month sera from mice immunized with K2-EPA or O1-EPA against various K2:O1 expressing *K. pneumoniae* strains: A) 43816, B) 736213, C) KR174, or D) 511348. Statistical analyses were performed via Mann-Whitney *U* tests comparing K2 and O1 percent survival at 1 month versus 6 month. The dotted line on each graph represents the point at which percent output is the same as percent input (100 % survival) *** *p* < 0.0001, **p < 0.001, ns: not significant. Error bars represent standard deviation.

After establishing that IgG antibodies were present and persisted up to six months after vaccination with our bioconjugate vaccines; we sought to determine if the antibodies remain functional as well. We utilized a serum bactericidal assay to determine the antibodies’ ability to induce complement-mediated killing of the four *K. pneumoniae* isolates. We tested murine sera from the 1-month and 6-month timepoints. As expected, we observed significant killing of the four isolates from the K2-EPA mouse sera with little to no difference in killing between the 1 month and 6-month sera (Fig. 3). We did observe a slight difference in K2-EPA killing between months 1 and 6 against 43816 and 511348. However, we observed little to no killing from the O1 sera against any of the four strains (Fig. 3). We observed minimal bactericidal activity against strain 511348 at 1 month, which was lost in the 6 month sera. This is consistent with previous observations of little O1 antibody killing by SBA, most likely due to capsular polysaccharide shielding O-specific immunoglobulins from reaching their target [5]. There also appears to be little difference between the 1-month and 6-month O1 sera (Fig. 3).

**Fig. 4.**
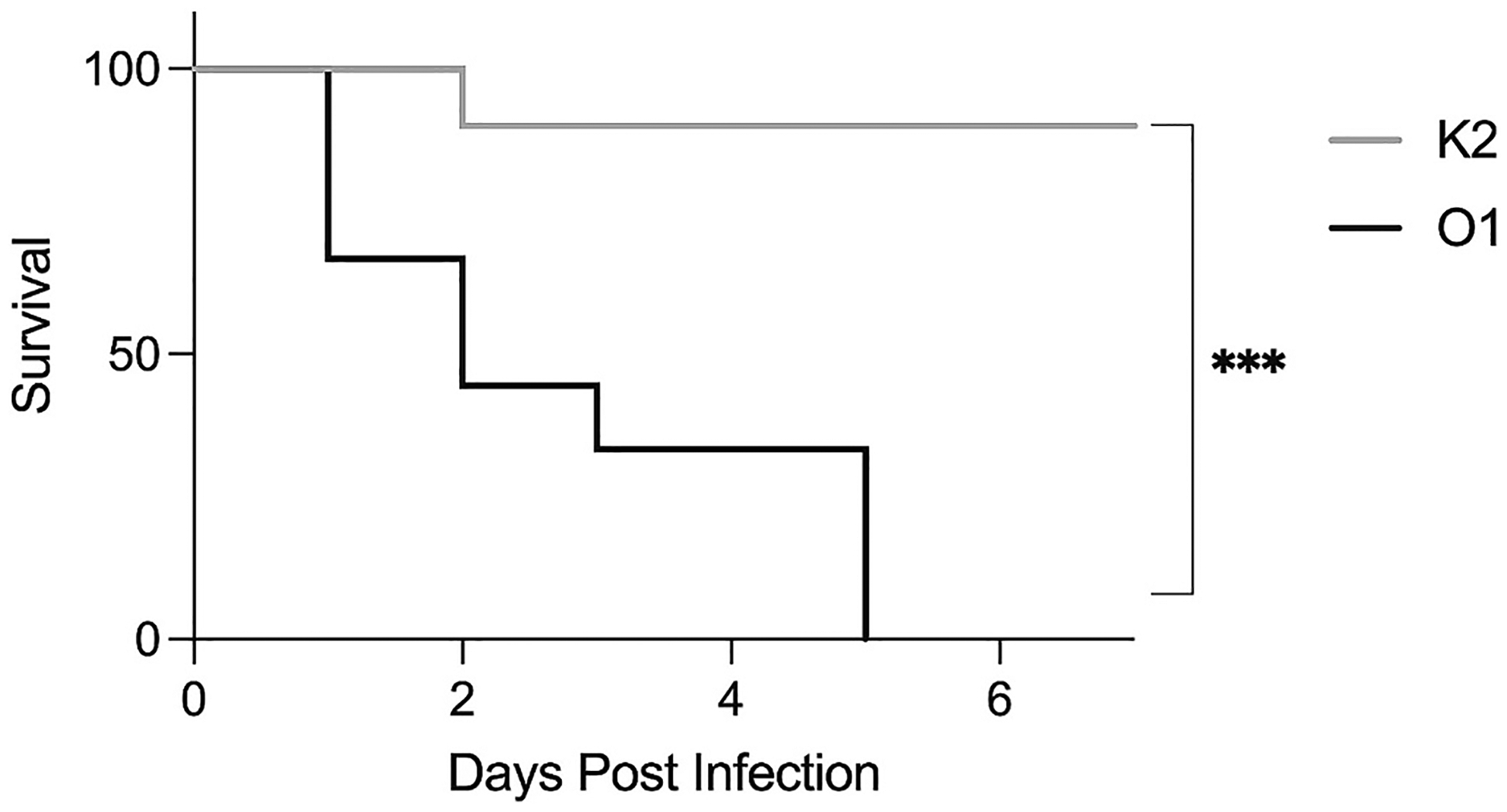
Lethal bacteremia challenge of mice 6-months after immunization. Mice were vaccinated with either K2-EPA or O1-EPA bioconjugate vaccines on days 0, 14, and 28 followed by bacteremia challenge via intraperitoneal injection with 43816 (2500 CFU) at 6 months post-immunization. Each group contains *n* = 10 mice combined male and female. Statistical analysis was performed via log-rank (Mantel-Cox) test. ***p < 0.001.

Finally, after demonstrating that there are long-term IgG antibodies present up to 6-months post-immunization with the *K. pneumoniae* bioconjugates and that at least a subset of these antibodies retain their functionality, we tested murine survival in a bacteremia challenge model. Mice that had undergone immunization 6-months prior were intraperitoneally injected with *K. pneumoniae* 43816 and monitored for survival over 7-days post-infection (Fig. 4). Mice that were immunized with the K2-EPA bioconjugate vaccine were significantly protected from infection compared to mice immunized with the O1-EPA bioconjugate. Importantly, these 6-month data are consistent with previous survival results from challenge 2-weeks post immunization [5]. Taken together, these data demonstrate that mice immunized with K2-EPA bioconjugate vaccine generate IgG antibodies that are long-lived through at least 6 months post-immunization. Further, these antibodies retain their functionality, demonstrating bacterial killing in vitro, and protect mice from a lethal bacteremia challenge.

## 4. Discussion

*K. pneumoniae* is becoming an increasingly difficult-to-treat pathogen as the rates of antibiotic resistance continue to rise. Vaccination represents a promising strategy to help combat this burden. However, there is currently not a licensed *K. pneumoniae* vaccine and only one formulation targeting four O-antigen polysaccharides is currently in clinical trials. Previous preclinical studies have suggested that capsular masking of O-antigens may limit the functionality of O-antibodies against some *K. pneumoniae* strains [5,6,8]. This work continues to emphasize this point across a wider timespan post-vaccination. We show evidence in all four K2:O1 isolates used in this study that O-antigen antibodies are masked from binding/killing these strains in ELISA and SBA experiments. Further, the O1 antibodies in O1-immunized mice failed to protect from lethal challenge with 43816. Capsule masking continues to be an important aspect to consider in *K. pneumoniae* vaccine design as an O-antigen-based vaccine may not protect against a wide variety of strains. We also noted a difference in the IgG subtypes elicited by each vaccine. Both groups of mice elicited robust IgG1 titers, generally the most abundant antibody subclass produced in response to vaccines and known to play a role in activating the complement cascade [9,10]. However, mice immunized with the K2-EPA vaccine elicited more IgG2b and IgG2c than mice immunized with the O1v1-EPA vaccine. This could be of interest as IgG2b plays important roles early on in Fc-mediated effector functions, complement activation, opsonization, and antibody-dependent cell-mediated cytotoxicity [9,10]. This could be another reason, beyond capsule masking, that O1v1 antibodies have a reduced ability to promote bacterial killing.

Here we describe evidence into the longevity (antibody persistence) and durability (antibody functionality) of the immune response elicited by two previously characterized bioconjugate vaccines. While the O1 vaccine appears to elicit antibodies that are long-lived, but not necessarily durable, the K2 vaccine antibodies appear both long-lived and relatively durable. The humoral response to the K2 capsule bioconjugate persists for at least six months in a murine model as evidenced by persistence of IgG antibodies, complement-mediated killing of bacteria by the antibodies, and survival in a lethal bacteremia challenge. We did observe statistically different bacterial killing against two strains of *K. pneumoniae* (43816 and 511348) between 1 month and 6 month serums. While there was a difference it is important to note that the difference between activity was slight, the antibodies were still able to induce bacterial killing, and that these antibodies were still durable enough at 6 months to protect the mice from 43816 bacteremia challenge. This work highlights the tremendous upside of conjugation: the capability of eliciting long-term immunity. Similar to other conjugate vaccines such as HiB and Prevnar [11,12], conjugate vaccines to *K. pneumoniae* capsular polysaccharides are also likely to provide persistent immune protection. As it is estimated that 15–25 capsular types would be required for broad protection in a multi-valent vaccine targeting *K. pneumoniae* [13], additional studies specifically assessing long-term monitoring of the immune response will be required as more antigens are added. However, the data presented here are an important first evidence of the tremendous longevity and durability associated with *K. pneumoniae* capsule bioconjugate vaccines.

## Data Availability

Data will be made available on request.
